# Immunoglobulin GM 3 23 5,13,14 phenotype is strongly associated with IgG1 antibody responses to *Plasmodium vivax *vaccine candidate antigens PvMSP1-19 and PvAMA-1

**DOI:** 10.1186/1475-2875-9-229

**Published:** 2010-08-09

**Authors:** Janardan P Pandey, Cristiane G Morais, Cor JF Fontes, Erika M Braga

**Affiliations:** 1 Department of Microbiology and Immunology, Medical University of South Carolina, Charleston, SC 29425, USA; 2 Departamento de Parasitologia, Universidade Federal de Minas Gerais, Belo Horizonte, Minas Gerais, Brazil; 3 Universidade Federal de Mato Grosso, Cuiabá, Mato Grosso, Brazil

## Abstract

**Background:**

Humoral immune responses play a key role in the development of immunity to malaria, but the host genetic factors that contribute to the naturally occurring immune responses to malarial antigens are not completely understood. The aim of the present investigation was to determine whether, in subjects exposed to malaria, GM and KM allotypes--genetic markers of immunoglobulin γ and κ-type light chains, respectively--contribute to the magnitude of natural antibody responses to target antigens that are leading vaccine candidates for protection against *Plasmodium vivax*.

**Methods:**

Sera from 210 adults, who had been exposed to malaria transmission in the Brazilian Amazon endemic area, were allotyped for several GM and KM determinants by a standard hemagglutination-inhibition method. IgG subclass antibodies to *P. vivax *apical membrane antigen 1 (PvAMA-1) and merozoite surface protein 1 (PvMSP1-19) were determined by an enzyme-linked immunosorbent assay. Multiple linear regression models and the non-parametric Mann-Whitney test were used for data analyses.

**Results:**

IgG1 antibody levels to both PvMSP1-19 and PvAMA-1 antigens were significantly higher (*P *= 0.004, *P *= 0.002, respectively) in subjects with the GM 3 23 5,13,14 phenotype than in those who lacked this phenotype.

**Conclusions:**

Results presented here show that immunoglobulin GM allotypes contribute to the natural antibody responses to *P. vivax *malaria antigens. These findings have important implications for the effectiveness of vaccines containing PvAMA-1 or PvMSP1-19 antigens. They also shed light on the possible role of malaria as one of the evolutionary selective forces that may have contributed to the maintenance of the extensive polymorphism at the GM loci.

## Background

Malaria is present in nearly 90 countries with approximately 2.5 billion people exposed to infection by *Plasmodium falciparum *and *Plasmodium vivax *[1]. Although causing less mortality than *P. falciparum*, *P. vivax *infection has an enormous socioeconomic impact. *P. vivax *is a widely distributed human malarial parasite, prevalent in South America, Asia and Oceania, and the 70-80 million cases currently recorded annually are of global public health importance [2]. *Plasmodium vivax *is now recognized as a cause of severe and fatal malaria, despite its low parasitaemia, the increased deformability of vivax-infected red blood cells and an apparent paucity of parasite sequestration [3]. The most cost-effective measure to control infectious diseases like malaria is a vaccine and effective malaria vaccines are still not available. Antigens of *Plasmodium *located on the surface or in the apical organelles of merozoites have been characterized as targets for protection or as possible vaccine antigens against malaria [4]. Among them, the apical membrane antigen 1 (AMA-1) and a 19-kDa fragment of merozoite surface protein-1 (MSP1-19) are the leading candidates for inclusion in a vaccine against blood stages of malaria. AMA-1 is an 83-kDa antigen synthesized during the mature stages of the parasite; it is thought to be involved in the process of erythrocyte invasion [4]. MSP1-19 is a portion of MSP1 produced after two processing steps and remains attached to the newly formed ring stage parasite after invasion [5]. Active immunization of experimental animals with either native or recombinant forms of both proteins has been shown to be protective against challenge infection [6]. Moreover, antibodies to MSP1-19 and AMA-1 inhibited invasion of red blood cells [7].

Humoral immune responses, which have a substantial genetic component [8], play a key role in the development of immunity to malaria. Identification and understanding of the mechanisms of action of host genetic factors that contribute to the naturally occurring anti-malarial immune responses is of utmost importance. The current paucity of knowledge in this area hinders effective immunological intervention and confounds the evaluation of ongoing vaccine efficacy trials. The few immune response genes identified thus far do not account for the total inter-individual variability in antibody responsiveness to malarial antigens [9,10], implying the involvement of additional genes. Immunoglobulin (Ig) allotypes are important candidates for controlling immune responsiveness, as evidenced by their association with humoral immunity to a variety of pathogens [11-16]. The role these polymorphic determinants play in antibody responses to malarial antigens, however, is not fully understood.

There are striking qualitative and quantitative differences in the distribution of Ig GM and KM allotypes among different ethnic groups [17,18]. Additionally, there is almost complete linkage disequilibrium between particular GM determinants within an ethnic group, and every major group is characterized by a distinct array of GM haplotypes. These population genetic properties suggest that differential selection over many generations may have played an important role in the maintenance of polymorphism at these loci, but the nature of putative evolutionary selective forces is not understood. As first suggested by J.B.S. Haldane, major infectious diseases like malaria, which probably coevolved with humans, have been the principal selective forces of natural selection [19]. One mechanism for how GM and KM determinants could contribute to the outcome of infection with various agents may be through allotype-restricted antibody responses to these pathogens, resulting in differential immunity to infectious diseases. The aim of the present investigation was to determine whether, in subjects exposed to malaria, GM and KM allotypes contribute to the magnitude of natural antibody responses to certain target antigens that are leading vaccine candidates for protection against *P. vivax*.

## Methods

### Subjects

The study population, derived from four sites and described in detail elsewhere [20,21], consisted of 210 non-infected (negative thick blood smears) adults who had been exposed to malaria hypo-mesoendemic transmission in the Brazilian Amazon area and reported a variable number of previous episodes caused by *P. vivax *or *P. falciparum *with clinical symptoms. The first group of subjects lived in Belém, the capital of Pará State. It was composed of 76 persons (median age, 29 years) who had acquired a single *P. vivax *malaria after a brief exposure (a few days) on the island of Cotijuba, which is located 25 km from Belém. The second group lived in Cuiabá, the capital of Mato Grosso, where no malaria transmission occurs. It was composed of 20 persons (median age, 33 years) who had become infected after short visits to mining and/or agricultural areas in the northern part of the state where malaria is endemic. They stayed less than one year in the transmission areas. The third group was composed of 65 persons (median age, 31 years) who had resided for more than 10 years in Terra Nova do Norte (TNN), a small rural community within the malaria endemic region. These individuals were continuously exposed to malaria transmission (median time, 10.5 years). The fourth group was composed of 49 persons who had lived for approximately 20 years in several gold-mining areas in the Brazilian Amazon basin. All lived in Apiacás, a municipality located in northern Mato Grosso State. Apiacás was characterized as a mesoendemic area because malaria prevalence in 1996, the study year, was 18%.

A complete health questionnaire was applied to all participants. Ethical clearance for this study was obtained from the Institutional Review Boards of the Federal University of Minas Gerais, Brazil and the Medical University of South Carolina, USA. Venous blood samples (2 mL) were drawn into EDTA containing tubes and used to obtain plasma which were frozen at -20°C for subsequent use.

### Antigens

Two recombinant proteins of *P. vivax *were employed in this investigation. Recombinant apical membrane antigen 1 (PvAMA-1), which contains the amino acid sequence 43-487 of the ectodomain of the *P. vivax *BEL-12 isolate, was expressed in *Escherichia coli *and purified as described previously [22]. The recombinant merozoite surface protein 1 (PvMSP1-19), which represents the amino acid sequence of the two EGF-like motifs of the Belem strain, was expressed in *Escherichia coli *in fusion with the glutathione S-transferase gene as described previously [23,24].

### Antibody determinations

IgG subclass determinations were made by an ELISA described elsewhere [20,21]. Briefly, the concentration of PvMSP1-19 and PvAMA-1 used were 0.5 μg/ml and 1.0 μg/mL, respectively. All samples were diluted 1:50. Mouse monoclonal antibodies to human IgG subclasses used were clone HP-6012 for IgG1, clone HP-6014 for IgG2, clone HP-6010 for IgG3, and clone HP-6025 for IgG4 (Sigma) diluted according to the manufacturer's specifications. Monoclonal antibody binding was detected with peroxidase conjugated anti-mouse immunoglobulin (Sigma). The threshold of positivity (cut-off value) was obtained by testing 40 different negative control sera from individuals not exposed to malaria from Belo Horizonte. The mean optical density value at 490 nm (OD490) ± 3 SD for duplicate determinations in negative sera was used as the cut-off value for different subclasses. The reactivity index (RI) was obtained to compare the levels of IgG subclasses among the subjects. The RI value was calculated by dividing the mean OD value for each test sample assayed by the cut-off value for each subclass tested using sera from healthy individuals (control group). Samples with an RI > 1 were considered positive.

### GM and KM allotyping

Serum samples were typed for G1 M (1/a, 2/x, 3/f, 17/z), G2 M (23/n), G3 M (5/b1, 6/c3, 13/b3, 14/b4, 21/g), and KM (1, 3) allotypes by a standard hemagglutination-inhibition method [25]. In brief, a mixture containing human blood group ORh+ erythrocytes coated with anti-Rh antibodies of known GM/KM allotypes, the test sera, and monospecific anti-allotype antibodies were incubated in a microtitre plate. Test sera containing IgG of particular allotype inhibited hemagglutination by the anti-allotype antibody, whereas negative sera did not. As mentioned before, linkage disequilibrium (allelic association) in the GM system is almost absolute and the determinants are transmitted as a group called haplotypes [26]. The notation follows the international system for human gene nomenclature [27], in which haplotypes and phenotypes are written by grouping together the markers that belong to each IgG subclass, by the numerical order of the marker and of the subclass; markers belonging to different subclasses are separated by a space, while allotypes within a subclass are separated by commas.

### Statistical analyses

Antibody levels against PvAMA-1 or PvMSP1-19 were described as median and interquartile range for all IgG subclasses. The allotype data were analysed as a group (phenotypes), rather than the presence or absence of individual markers, as the significant linkage disequilibrium in this system may be the result of certain selective evolutionary advantages (against immunity to pathogens like malaria), making the analysis by phenotypes biologically more meaningful. Subjects with very unusual GM phenotypes and those whose frequency was <3% were combined as "other" for statistical analyses, so as not to have a test with too many degrees of freedom or categories with cell counts too small for appropriate analyses. This method of grouping increases statistical power and makes use of all data. In univariate analyses, the non-parametric Mann-Whitney test was used to determine whether GM and KM phenotypes were associated with anti-*P. vivax *IgG isotype-specific antibody levels (reactivity index). Subsequently, multiple linear regression models were used to investigate the independent contribution of cumulative exposure to malaria (according to the study site) to GM and KM phenotype distribution and antibody responses. Thus, phenotypes associated with levels of antibodies against PvMSP1-19 and PvAMA-1 using the Mann-Whitney univariate analyses were also included in multiple linear regression analysis by study sites. In all models, statistical significance was defined as P < 0.05.

## Results

The distribution of GM and KM phenotypes in relation to IgG subclass antibody levels to PvMSP1-19 and PvAMA-1 is given in Tables 1 and 2, respectively. Of the entire study population, 26 subjects could not be characterized for antibodies to PvMSP1-19, reflecting the differences in the total number of individuals in the two tables. The majority of the GM phenotypes observed could be explained by postulating the segregation of common Caucasoid (GM 1,17 21, GM 1,2,17 21, GM 3 5,13,14 and GM 3 23 5,13,14) and Negroid (GM 1,17 5,6, GM 1,17 5,14, GM 1,17 5,6,14, and GM 1,17 5,13,14) haplotypes.

**Table 1:** GM/KM phenotypes in relation to IgG subclass-specific antibodies to PvMSP1-19 in subjects exposed to malaria

Phenotypes (Number of subjects)	Antibody levels (Reactivity Index) (Median, Interquartile range)			
	
	IgG1	IgG2	IgG3	IgG4
GM 1,2,17 21 (9)	0.63(0.01-2.67)	0.05(0.01-0.21)	0.35(0.01-1.53)	0.01(0.01-0.18)
GM 1,17 5,13,14,21 (15)	0.87(0.55-1.97)	0.29(0.01-0.82)	1.66(0.01-6.31)	0.44(0.01-0.58)
GM 1,2,3,17 23 5,13,14,21 (13)	2.65(0.32-4.26)	0.33(0.01-0.46)	3.17(0.44-5.25)	0.11(0.01-0.48)
GM 1,3,17 23 5,13,14,21 (28)	0.76(0.01-3.57)	0.11(0.00-0.40)	^d^0.15(0.01-2.74)	0.09(0.01-0.52)
GM 1,3,17 23 5,6,13,14 (10)	1.88(1.49-3.37)	0.16(0.02-0.50)	0.47(0.02-2.40)	0.09(0.01-0.23)
GM 1,3,17 5,13,14,21 (18)	2.55(0.21-4.07)	0.31(0.06-1.54)	1.68(0.01-4.46)	^f^0.30(0.01-0.99)
GM 3 23 5,13,14 (20)	^b^3.60(1.89-4.71)	0.16(0.03-1.25)	2.49(0.25-5.81)	0.08(0.01-0.58)
^a^Other GM (71)	2.27(0.25-3.31)	0.28(0.01-0.67)	1.44(0.08-5.06)	0.17(0.01-0.43)
KM 1 (9)	1.18(0.01-2.44)	^c^0.02(0.01-0.16)	^e^0.01(0.01-2.35)	0.01(0.01-0.46)
KM 1,3 (68)	1.75(0.53-3.48)	0.18(0.00-0.64)	1.44(0.13-5.81)	0.19(0.01-0.56)
KM 3 (107)	2.32(0.18-3.86)	0.22(0.01-0.68)	1.53(0.06-3.83)	0.13(0.01-0.40)

^a^Other GM [(GM 1,17 21 (n = 5), GM 1,17 5,6,14,21 (n = 5), GM 3 5,13,14 (n = 4), GM 1,17 13,21 (n = 5), GM 1,17 6,14,21 (n = 1), GM 1,2,17 13,21 (n = 4), GM 1,2,17 13,14,21 (n = 2), GM 1,3,17 13,14,21 (n = 1), GM 1,17 23 5,13,14,21 (n = 1), GM 1,17 5,6,13,14 (n = 4), GM 1,17 5,6,13,14,21 (n = 4), GM 1,17 5,14,21 (n = 2), GM 1,2,17 5,6,13,14,21 (n = 1), GM 1,2,17 5,6,14,21 (n = 4), GM 1,2,17 5,6,21 (n = 2), GM 1,2,3,17 5,6,13,14,21 (n = 1), GM 1,3,17 23 5,6,13,14,21 (n = 5), GM 1,3,17 5,13,14 (n = 4), GM 1,3,17 5,6,13,14 (n = 5), GM 1,3,17 5,6,13,14,21 (n = 3), GM 1,2,17 5,13,14,21 (n = 3), GM 1,2,3,17 5,13,14,21 (n = 5)]

^b^significantly higher (*P *= 0.004), ^c^significantly lower (*P *= 0.031), ^d^significantly lower (*P *= 0.051),

^e^significantly lower (*P *= 0.027), ^f^significantly higher (*P *= 0.002), (obtained by Wilcoxon-Mann-Whitney test) compared with the rest of the GM phenotypes.

**Table 2:** GM/KM phenotypes in relation to IgG subclass-specific antibodies to PvAMA-1 in subjects exposed to malaria

Phenotypes (Number of subjects)	Antibody levels (Reactivity Index) (Median, Interquartile range)			
	
	IgG1	IgG2	IgG3	IgG4
GM 1,2,17 21 (10)	^b^0.15(0.10-0.48)	0.02(0.01-0.62)	0.57(0.18-1.04)	0.06(0.01-0.64)
GM 1,17 5,13,14,21 (21)	^c^0.28(0.05-0.58)	0.4(0.05-1.21)	0.47(0.24-1.05)	0.37(0.05-0.75)
GM 1,2,3,17 23 5,13,14,21 (14)	1.09(0.54-3.70)	0.42(0.16-0.88)	0.45(0.26-0.73)	0.23(0.03-0.38)
GM 1,3,17 23 5,13,14,21 (33)	^d^2.02(0.61-3.67)	0.50(0.16-1.23)	0.66(0.31-1.72)	0.58(0.13-0.93)
GM 1,3,17 23 5,6,13,14 (10)	1.68(0.22-3.83)	0.01(0.01-0.30)	0.31(0.01-1.10)	0.60(0.01-0.91)
GM 1,3,17 5,13,14,21 (20)	0.94(0.18-3.98)	0.35(0.01-0.92)	0.47(0.09-1.86)	0.27(0.06-0.92)
GM 3 23 5,13,14 (22)	^e^3.81(1.18-11.78)	0.45(0.01-1.71)	0.76(0.12-1.32)	0.15(0.01-2.30)
^a^Other GM (80)	^f^0.58(0.11-2.09)	0.39(0.13-1.66)	0.59(0.18-1.09)	0.32(0.03-0.87)
KM 1 (11)	0.71(0.03-1.10)	0.13(0.01-0.44)	0.28(0.10-0.82)	0.47(0.38-0.95)
KM 1,3 (80)	0.67(0.27-1.96)	0.49(0.14-1.09)	0.57(0.23-1.06)	0.33(0.04-0.69)
KM 3 (119)	0.94 (0.19-3.69)	0.37(0.01-1.06)	0.57(0.21-1.14)	0.29(0.03-0.85)

^a^Other GM [(GM 1,17 21 (n = 5), GM 1,17 5,6,14,21 (n = 5), GM 3 5,13,14 (n = 4), GM 1,17 13,21 (n = 5), GM 1,17 6,14,21 (n = 1), GM 1,2,17 13,21 (n = 5), GM 1,2,17 13,14,21 (n = 3), GM 1,3,17 13,14,21 (n = 2), GM 1,17 23 5,13,14,21 (n = 2), GM 1,17 5,6,13,14 (n = 5), GM 1,17 5,6,13,14,21 (n = 4), GM 1,17 5,14,21 (n = 2), GM 1,2,17 5,6,13,14,21 (n = 2), GM 1,2,17 5,6,14,21 (n = 4), GM 1,2,17 5,6,21 (n = 2), GM 1,2,3,17 5,6,13,14,21 (n = 1), GM 1,3,17 23 5,13,14 (n = 1), GM 1,3,17 23 5,6,13,14,21 (n = 5), GM 1,3,17 5,13,14 (n = 5), GM 1,3,17 5,6,13,14 (n = 5), GM 1,3,17 5,6,13,14,21 (n = 3), GM 1,2,17 5,13,14,21 (n = 4), GM 1,2,3,17 5,13,14,21 (n = 5)]

^b^significantly lower (*P *= 0.019), ^c^significantly lower (*P *= 0.0003), ^d^significantly higher (*P *= 0.007), ^e^significantly higher (*P *= 0.002), ^f^significantly lower (*P *= 0.029) (obtained by Wilcoxon-Mann-Whitney test) compared with the rest of the GM phenotypes.

IgG1 antibody responses to both PvMSP1-19 (Table 1) and PvAMA-1 (Table 2) were highly significantly associated with the GM 3 23 5,13,14 phenotype. For PvMSP1-19, carriers of the GM 3 23 5,13,14 phenotype had higher levels of IgG1 than noncarriers (median [interquartile age, IQR]: 3.60 RI [1.89-4.71] versus 1.59 RI [0.18-3.36], *P *= 0.004). This association was even more pronounced for antibody responses to PvAMA-1: carriers of the GM 3 23 5,13,14 phenotype had higher levels of IgG1 than noncarriers: 3.81 RI [1.18-11.78] versus 0.67 RI [0.20-2.13], *P *= 0.002). No significant interactions between GM and KM phenotypes were found for antibody responsiveness to either PvAMA-1 or PvMSP1-19 antigens. As presented in the footnotes of Tables 1 and 2, the results of the univariate analyses showed several significant associations between GM and KM phenotypes and antibody responsiveness, but after analyses using the multiple linear regression models and controlling for the study sites (different levels of cumulative exposure to malaria), only the GM 3 23 5,13,14 phenotype showed an independent and positive association with the level of IgG1 anti-malarial antibodies (regression coefficient 1.03 [95% CI 0.13-1.93], *P *= 0.025 and regression coefficient 4.56 [95% CI 2.78-6.34], *P *= 0.000 for PvMSP1-19 and PvAMA-1, respectively).

## Discussion

A number of studies have shown that anti-malarial antibodies of IgG1 subclass contribute significantly to protective immunity against malaria [28,29]. A major finding of the present investigation is that IgG1 antibody levels to both PvMSP1-19 and PvAMA-1 antigens were significantly higher in subjects with the GM 3 23 5,13,14 phenotype than in those who lack this phenotype. Several mechanisms could account for these observations. One mechanism might involve GM allotypes being part of the recognition structures for PvMSP1-19 and PvAMA-1 antigens. Memory B cells, which predominantly express IgG as the membrane-bound form of Ig, show enhanced response to antigen stimulation than cells expressing IgM on their surface [30,31]. Perhaps membrane-bound IgG molecules with the GM 3 23 5,13,14 phenotype are more efficient in the uptake, processing, and subsequent presentation of PvMSP1-19 and PvAMA-1 epitopes to the collaborating T cells, resulting in strong humoral immunity.

Additionally, GM allotypes could contribute to antibody responsiveness through their direct or indirect involvement in antibody specificity. There is growing body of evidence for the involvement of these constant-region determinants in antibody specificity usually associated with the variable region of the Ig molecule [32]. Possible mechanisms include direct contribution to the formation of idiotypic determinants, modulation of antibody binding affinity, and linkage disequilibrium with alleles coding for the variable-region epitopes [33-36].

The possibility of GM allotype involvement in malarial immunity was suggested by the results of a previous investigation, which showed significant differences in the frequencies of these determinants between two African sympatric tribes--Fulani and Masaleit--with marked differences in susceptibility to *P. falciparum *malaria [37]. A recent study has reported significant associations between particular GM phenotypes and clinical presentation of *P. falciparum *malaria in Beninese children [38]. In a population from Sudan, GM phenotypes were found to be associated with susceptibility to *P. falciparum *malaria and with antibody responsiveness to malarial antigens [39]. The Sudanese study, however, is not comparable to the present study employing subjects from Brazil. In addition to the differences in the pathogen (*P*. *falciparum *vs *P*. *vivax*), there are highly significant differences in the frequency of GM/KM allotypes between the two populations (26). For instance, none of the Sudanese subjects possessed the GM 3 23 5,13,14 Phenotype, which is associated with antibody responsiveness in the present study, underscoring the necessity of studying different populations.

Because malaria is a major global health problem, there is clear urgency to develop a vaccine for protection against infection. Development of an effective vaccine, however, would require a thorough understanding of the mechanisms associated with immunity to this parasite. Furthermore, given the complexity of the malarial parasite and tremendous genetic diversity of human populations, it may be difficult to devise one vaccine that is effective against all variants of the parasite and provides protective immunity for all people. Studies of the kind reported here might be able to identify people who are more likely to respond to a given vaccine. For the subjects with non-responders genotypes, the vaccine could be fused with appropriate adjuvants to circumvent the allotypic restriction in immune responsiveness. There is precedence for converting genetic non-responders into responders to a given vaccine. For instance, subjects classified as non-responders to *Haemophilus influenzae *type b polysaccharide vaccine--based on their GM 23 and KM 1 allotype status--became responders when the vaccine was conjugated to a suitable adjuvant [40-42]. The mechanism underlying the circumvention of allotype-associated differences in antibody responses is not understood. Ability to identify potential responders to a given vaccine would be considerably enhanced by simultaneous examination of other genes of the immune system, such as HLA, FcγRIIa, FcγRIIIa, TNF, which have been shown to make significant contributions to malarial immunity. Genes do not act in isolation: there is growing body of evidence that epistasis--modification of the action of a gene by one or more other genes--plays a significant role in human diseases [43]. Therefore, it is important to investigate the role of possible allelic associations between candidate genes in influencing various parameters of immunity to malaria. For instance, GM allotypes may influence antibody-dependent cellular inhibition (ADCI) and phagocytosis of malarial parasites by their differential interaction with FcγR expressed on effector cells. The majority of GM epitopes are present on the Fc portion of the IgG molecule. Thus, Fc of a particular GM genotype could preferentially bind to the FcγRIIa or FcγRIIIa of a particular genotype and influence immunity to malaria through ADCI, which is one of the most efficient mechanisms through which antibodies protect against malaria. This preferential ligand-receptor interaction would be analogous to the reported interaction between particular killer cell immunoglobulin-like receptors and their HLA-C ligand in the resolution of hepatitis C virus infection [44]. Such studies would require large study populations and collaboration of several research centers.

Although plausible mechanisms of direct involvement of GM allotypes in humoral immunity to malaria are discussed here, the associations observed in this study could also result from linkage disequilibrium between as-yet-unidentified putative immune response gene(s) for PvMSP1-19 and PvAMA-1 antigens and particular GM alleles. Therefore, it is important to expand these studies to include other ethnic groups, characterized by a different array of GM haplotypes, resulting from ethnic differences in linkage disequilibrium among GM alleles [26]. This is the first report of association between GM allotypes and antibody responsiveness to *P. vivax *malarial antigens.

## Conclusions

Anti-malarial antibodies of IgG1 subclass have been previously shown to contribute significantly to protective immunity against malaria. Results presented here show that IgG1 antibody levels to *P. vivax *PvMSP1-19 and PvAMA-1 antigens were significantly higher in subjects with the GM 3 23 5,13,14 phenotype than in those who lacked this phenotype. If these findings were confirmed in an independent study, they could aid in identifying subjects (GM 3 23 5,13,14) who are more likely to benefit from PvMSP1-19 and PvAMA-1 containing vaccines. For the non-carriers of GM 3 23 5,13,14, these antigens could be fused with appropriate adjuvants to circumvent the allotypic restriction in immune responsiveness.

The nature of the evolutionary selective mechanism(s) that maintains polymorphisms at the GM loci is not known. Strong associations between GM allotypes and antibody levels to malarial antigens suggest that malaria may have been one of the selective forces that have contributed to the maintenance of the balanced polymorphism at these loci.

## Competing interests

The authors declare that they have no competing interests.

## Authors' contributions

JP conceived the study. All authors participated in the acquisition of data. EB participated in the design of the study and performed the statistical analysis. JP and EB were responsible for data interpretation and drafting the manuscript. All authors read and approved the final manuscript.
